# Immune Privilege and the Philosophy of Immunology

**DOI:** 10.3389/fimmu.2014.00110

**Published:** 2014-03-19

**Authors:** Joan Stein-Streilein, Rachel R. Caspi

**Affiliations:** ^1^Department of Ophthalmology, Schepens Eye Research Institute, Mass Eye and Ear, Harvard Medical School, Boston, MA, USA; ^2^Laboratory of Immunology, National Eye Institute, National Institutes of Health, Bethesda, MD, USA

**Keywords:** immune privilege, tolerance, immune suppression, eye, regulatory cells

Like other scientists, immunologists use two types of approaches to research: one reduces the problem to its parts; the other studies the emergent phenomenon produced by the parts. Scientists that reduce the problem to its parts are sometimes called reductionists. The conclusions of reductionist experiments are often applied to the greater whole, when in actuality they may only apply to that particular experimental set. We, reductionists, are the ones who think our immune behavior exists solely because of genes, the presence of TGFβ, the presence of inflammatory cytokines, and appearance of a receptor. We tend to interpret the outcome in the colors of our interests (*ergo* “to a hammer, everything is a nail”). We measure parts and from the parts, we draw parallels to far-removed outcomes in terms of health and disease. More often than not, however, the results from the study of the parts do not predict the whole, and often the whole becomes greater than the sum of its parts. For example, a toll-like receptor does one thing when there is a bacterial invasion but the same toll-like receptor may lead to a different outcome when activated by danger signals during injury.

The approach that is perhaps more applicable to biology, and more specifically, to immunology, is the chaos theory. The chaos theory deals with the multiple layers of conditions and unexpected turns and restarts that can effect the outcome. It is applicable to studies of dynamic systems like the weather, and in our case, dynamic biological/immunological systems. The chaos theory points out that small differences in initial conditions make it impossible to predict the outcome. Thus, the behavior of the parts does not make the outcome predictable.

Our point? Immune privilege is broadly understood as the ability of the tissue to actively regulate and direct immune responses that take place in its “territory.” The articles in this Research Topic in Frontiers in Immunology, “Good news–bad news”, support the idea that to understand how immune privilege works, one has to understand the dynamics of the different tissues in terms of initiation, expression, regulation, and behavior, before one can begin to predict an outcome.

This Research Topic contains a number of papers that deal with immune privilege in the eye: a review that explores the relationship of immune privilege to ocular disease (1); a contribution on local regulation of immune CD8^+^ T cells in the eye (2); a review discussing the intriguing parallels between the mechanisms of ocular immune privilege and uveal melanoma (3); a thoughtful contribution on how CD8^+^ Treg cells might enable ocular tumor growth (4); a review proposing that (paradoxically) immune privilege emerges as an enabler of immune cells that heal, as well as of regulator immune cells that promote tissue damage (5).

While the eye is considered the prototypic immune privileged tissue, it is not the only one. We therefore shift to reviews of immune privilege and its mechanisms in other tissues and organs, underscoring the universality of the phenomenon: the reproductive tract (6), the testis (7), tumor environment (8), and finally chronic inflammatory diseases (9). While the eye, testes, reproductive tract, tumors, and chronic immune diseases all seem to share some “immune privileged” mechanisms, each has developed unique features of its own. Recent reports have repeatedly shown that immune regulation is “tailored” to the individual tissue in which it is taking place.

Each immune privileged tissue has a unique function. The eye must protect the light path and signals that stimulate the retina, and photoreceptors to preserve vision. The testis has to protect the sperm as they proceed to the epididymis where they mature. The maternal reproductive tract has to protect its eggs both before and after fertilization and thus has developed specialized mechanisms to modify the body’s response to foreign antigens. These unique challenges require different solutions and lead to unique immune privilege mechanisms. However, although microenvironment and the stromal cells that carry out the particular function may vary between tissues, and consequently the mechanisms that promote regulation may be unique to that cell or tissue, the goal is the same: limit collateral damage to preserve tissue integrity and maintain homeostasis to the extent possible, without compromising host defense.

Many of the studies in the tissues other than the eye reveal areas of investigations that have not been well studied in the eye. IDO exerts profound effects on immune and tissue cells that suppress pro-inflammatory and immune stimulatory responses to a variety of insults. While the eye does not have frequent exposure to foreign antigens, its many layers of immune regulation appears to allow unmatched cornea grafts long-term survival without systemic immunosuppression. It is clear that we do not know all the immune privilege paradigms, but by understanding the emergent phenomena that are produced by the parts of immune privilege that is used by other tissues, may help to understand the concept of immune privilege as a whole. The needs of the tissue, its environment, and consequently the mechanisms used by each tissue to immunoregulate may vary. But yet, many of the basic concepts may be shared. Thus, out of the parts, emerges a whole that is greater than the sum of the parts.

We hope this Research Topic has successfully outlined the many layers involved in immune privilege, established that they vary with each tissue and clarified that the outcome cannot always be predicted. Because clinical expectations of medical research often hurry scientific discoveries prematurely to therapeutic applications, the scientists who break new ground should also use caution. Caution, that premature application of the basic science *good news* without sufficient understanding and application of the chaos theory, may lead to *bad news*.
